# Cost-effectiveness of artificial intelligence screening for diabetic retinopathy in rural China

**DOI:** 10.1186/s12913-022-07655-6

**Published:** 2022-02-25

**Authors:** Xiao-Mei Huang, Bo-Fan Yang, Wen-Lin Zheng, Qun Liu, Fan Xiao, Pei-Wen Ouyang, Mei-Jun Li, Xiu-Yun Li, Jing Meng, Tian-Tian Zhang, Yu-Hong Cui, Hong-Wei Pan

**Affiliations:** 1 Department of Ophthalmology, the First Affiliated Hospital, Jinan University, Guangzhou, China; 2 Institute of Ophthalmology, School of Medicine, Jinan University, Guangzhou, China; 3 Department of Public Health and Preventive Medicine, School of Medicine, Jinan University, Guangzhou, China; 4 Department of Ophthalmology, Affiliated Hospital of Weifang Medical University, Weifang, China; 5 College of Pharmacy, Jinan University, Guangzhou, China; 6 School of Basic Medical Sciences, The Guangzhou Institute of Cardiovascular Disease, The Second Affiliated Hospital, Guangzhou Medical University, Guangzhou, China; 7 Department of Histology and Embryology, School of Basic Medical Sciences, Guangzhou Medical University, Guangzhou, China

**Keywords:** Diabetic retinopathy, Screening, Artificial intelligence, Cost-effectiveness, Markov model

## Abstract

**Background:**

Diabetic retinopathy (DR) has become a leading cause of global blindness as a microvascular complication of diabetes. Regular screening of diabetic retinopathy is strongly recommended for people with diabetes so that timely treatment can be provided to reduce the incidence of visual impairment. However, DR screening is not well carried out due to lack of eye care facilities, especially in the rural areas of China. Artificial intelligence (AI) based DR screening has emerged as a novel strategy and show promising diagnostic performance in sensitivity and specificity, relieving the pressure of the shortage of facilities and ophthalmologists because of its quick and accurate diagnosis. In this study, we estimated the cost-effectiveness of AI screening for DR in rural China based on Markov model, providing evidence for extending use of AI screening for DR.

**Methods:**

We estimated the cost-effectiveness of AI screening and compared it with ophthalmologist screening in which fundus images are evaluated by ophthalmologists. We developed a Markov model-based hybrid decision tree to analyze the costs, effectiveness and incremental cost-effectiveness ratio (ICER) of AI screening strategies relative to no screening strategies and ophthalmologist screening strategies (dominated) over 35 years (mean life expectancy of diabetes patients in rural China). The analysis was conducted from the health system perspective (included direct medical costs) and societal perspective (included medical and nonmedical costs). Effectiveness was analyzed with quality-adjusted life years (QALYs). The robustness of results was estimated by performing one-way sensitivity analysis and probabilistic analysis.

**Results:**

From the health system perspective, AI screening and ophthalmologist screening had incremental costs of $180.19 and $215.05 but more quality-adjusted life years (QALYs) compared with no screening. AI screening had an ICER of $1,107.63. From the societal perspective which considers all direct and indirect costs, AI screening had an ICER of $10,347.12 compared with no screening, below the cost-effective threshold (1–3 times per capita GDP of Chinese in 2019).

**Conclusions:**

Our analysis demonstrates that AI-based screening is more cost-effective compared with conventional ophthalmologist screening and holds great promise to be an alternative approach for DR screening in the rural area of China.

## Background

Diabetic retinopathy (DR) is one of the most important microvascular complications of diabetes, which is difficult to be detected until irreversible damage or even blindness occurs [1–3]. DR was ranked fifth among the major causes of global blindness [4]. The number of diabetic patients is projected to increase to 600 million by 2040, with one third expected to have diabetic retinopathy [5–7], presenting a huge medical and economic burden worldwide, especially in developing countries such as China. As reported by International Diabetes Federation diabetes atlas, there are 113.9 million adults with diabetes in China, which accounts for about 24% of all diabetic patients worldwide. Presently about half of the population, approximately 700 million people, are living in the rural areas of China, where the prevalence of diabetic retinopathy is higher than that of urban areas [8]. However, DR screening is not well performed in the rural areas of China due to unaffordability of medical cost, lack of medical facilities and limited access to conventional screening programs. Considering the importance of regular screening of people with diabetes for timely intervention and reduction of vision impairment [9], it is urgent to take measures to make DR screening more available and affordable in the rural areas of China. In this aspect, a new kind of screening strategy for DR incorporating artificial intelligence technology has great potential, especially in low- and middle-income countries [10].

Artificial intelligence (AI) using deep learning systems (DLS) emerges as a promising alternative approach in medical diagnosis of a variety of diseases, including diabetic retinopathy. It can provide instant DR diagnosis and reduce the burden of health system [11]. A DLS developed in Singapore has shown comparable diagnostic performance to human assessors and the savings to Singapore health system associated with switching the human assessment model to the semi-automated model are estimated to be $489,000, only 20% of the current annual screening cost [12, 13]. In India, an AI algorithm for DR and vision-threatening DR (VTDR) detection, using Remidio Fundus, produced a sensitivity of 96% and a specificity of 80% in detecting any diabetic retinopathy as well as a sensitivity of 99% and a specificity of 80% in detecting VTDR [14]. In China, the performance of a DLS model was evaluated for screening pre-proliferative diabetic retinopathy and diabetic macular edema, and the results showed a sensitivity of 97% and a specificity of 91% based on 19,900 images [14]. It has been found feasible to carry out AI based screening for DR in community hospitals [15]. As the study conducted in Xinjiang, China showed, AI had the same specificity (100%) and higher sensitivity (100% vs 79.1%) for referral DR screening, compared with manual screening [16]. It has been shown AI had relatively good consistency with ophthalmologist in DR grading, high specificity and acceptable sensitivity for the diagnosis of referral DR and any DR in community of China [17]. In Spain, AI system showed acceptable sensitivity (100% for referral DR and VTDR) and specificity (81.82% for referral DR and 94.64% for VTDR) against manual grading as well [18]. Compared with the conventional screening programs, AI screening can address several barriers including availability of human assessors, long-term financial sustainability and the growing need for DR screening and monitoring [13, 19].

To date, good diagnostic consistency in DR has been demonstrated between AI and manual grading. But little is known about the cost-effectiveness for AI based DR screening in rural China or other countries [20]. In order to provide economic evidence for medical and healthcare decision making, we assessed the cost-effectiveness of AI screening for DR relative to the conventional screening strategies using a Markov model, from the health system and societal perspectives.

## Methods

### Study Setting and description

The study was set in rural China. We conducted the analysis with a hypothetic cohort of 1000 patients in rural China. All patients were newly diagnosed with diabetes but without diabetic retinopathy, whose mean starting age was 44 years, representing the actual age distribution of patients with diabetes in rural China [21]. They were allowed to enter in one of the three screening groups: no screening group (the baseline group), AI screening group or ophthalmologist screening group, which meant they would take the DR screening and follow-up examinations later in the corresponding way. It was simulated in 35 yearly cycles. Rural China is defined as an area inhabited mainly by agricultural population engaged in agricultural production in China. According to the "Regulations for the Compilation of Statistical Division Codes and Urban–Rural Division Codes" formulated by the National Bureau of Statistics, the urban–rural division code is used to confirm whether the area is urban or rural (available at http://www.stats.gov.cn/tjsj/tjbz/tjyqhdmhcxhfdm/). The urban–rural division code starting with 1 indicates that it is urban while the code starting with 2 indicates that it is rural. The ophthalmologists, the staff and the patients in DR screening, were aware of the research program and we have given their written informed consent. For conventional screening of DR in rural areas, medical teams with facilities and computational resources would go to the community health service stations in rural areas and perform screening. The medical staff would complete fundus images capture and visual acuity tests. The Ophthalmologists then would grade the fundus images combined with the results of the vision examination. Patients identified with vision-threatening DR (VTDR), including severe non-proliferative diabetic retinopathy (NPDR) and proliferative diabetic retinopathy (PDR), would be referred to superior hospitals to get laser treatment. Those identified with no diabetic retinopathy (NO DR) and mild diabetic retinopathy (Mild DR) would be recalled for a follow-up examination every year in superior hospitals while those with moderate diabetic retinopathy (Moderate DR) would be recalled for a follow-up examination every half year in superior hospitals. All of the follow-up examination’s results would be graded by ophthalmologists. The follow-up examinations included taking fundus images, performing a screening visual acuity exam, an intraocular pressure examination and a slit lamp microscope examination [22]. For AI screening, medical teams with facilities and computational resources would go to the community health service stations in rural areas and perform screening as well. An AI-based software would be used to grade fundus images instead of ophthalmologists. After the fundus images are obtained and vision examinations are performed, the AI-based software would be applied to grade the fundus images quickly and accurately, as well as giving management advice. The recommendations for patients in different DR progressions would be the same as ophthalmologist screening. What is different is that all the results of the follow-up examinations would be graded by the AI-based software. The AI-based software had very similar sensitivity and specificity to ophthalmologists but with less cost and time for grading [23, 24].

### Model design

We developed a hybrid decision tree based on Markov model to analyze the costs, effectiveness of each screening strategy. We also calculated the incremental cost-effectiveness ratio (ICER) of AI screening strategies relative to no screening strategies over 35 years. The model was developed with Treeage pro 2021 (TreeAge Software Inc, Williamstown, MA, USA), running for 35 cycles according to the mean life expectancy of patients diagnosed with diabetes in rural China [21].

**Fig. 1:**
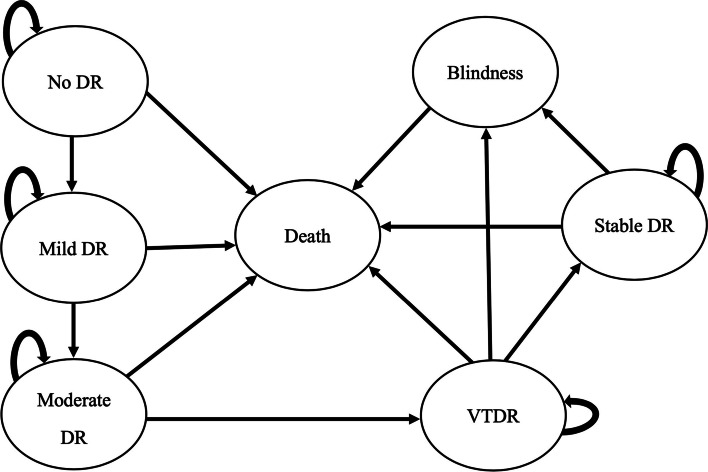
Markov Model structure. DR=diabetic retinopathy; VTDR= vision-threatening diabetic retinopathy

The model simulated the progression of DR after being diagnosed with diabetes. The Markov model for diabetic retinopathy was based on the Early Treatment Diabetic Retinopathy Study (ETDRS) criteria [25], in which the patients were classified into seven health states: No DR, Mild DR, Moderate DR, VTDR, Stable DR, Blindness and death. In each cycle (every single year), the transition allowed between health states was as follows: No DR may remain or progress to mild DR. Mild DR may remain or progress to moderate DR. The Moderate DR may remain or progress to VTDR. Patients diagnosed with VTDR needed to receive laser treatment. If the treatment succeeded, the health state would stay at Stable DR, which was recalled for a follow-up examination every year. If failed, it would stay VTDR, which might progress to blindness. Meanwhile, Stable DR may remain or progress to blindness as well. People with all health states were likely to die, which was related to their age instead of DR progression. The Markov model structure was shown in Fig. 1.

### Model inputs

Utility values (effectiveness) and transition probabilities were derived from literature. Mortality risks were calculated by multiplying the age-specific mortality risks by the mortality multipliers for diabetes and blindness, using linear interpolation. The natural age specific rates of mortality were derived from Chinese researchers [26]. Grading accuracy (sensitivity and specificity) of the two screening strategies was obtained from two published papers [23, 24], as shown in Table 1. Primary data was collected on the costs of ophthalmologist screening, AI screening and laser treatment, as shown in Table 2. Screening and treatment costs were collected from the Affiliated hospital of Weifang Medical University. AI software fee was obtained from the market quotation of the AI software supplier.

**Table 1:** Markov Model Parameter Estimates and Assumptions

Parameter	Value	Sensitivity Analysis Range
1. DR transition probabilities		
No to Mild [27]	0.07	0.01–0.10
Mild to Moderate [27]	0.19	0.166–0.214
Moderate to VTDR [27]	0.17	0.147–0.193
VTDR to Stable [28]	0.90	0.881–0.919
Stable to Blindness [29]	0.02	0.002–0.03
VTDR to Blindness [29]	0.09	0.07–0.11
2. Utility		
No DR [30]	0.94	0.83–1.05
Mild DR [30]	0.87	0.73–1.01
Moderate DR [30]	0.87	0.73–1.01
VTDR [30]	0.83	0.74–0.92
Stable DR [31]	0.85	0.72–0.78
Blindness [30]	0.81	0.73–0.89
3. Disutility of DR [32]	0.066	-
4. Mortality multipliers [26]		
Blindness	2.34	2.22–2.46
Diabetes	1.90	1.04–2.7
5. Sensitivity, %		
AI Screening screening [23]	90.79	86.40-94.10
Ophthalmologist screening [24]	96.00	94.79-97.21
6. Specificity, %		
AI screening [23]	98.50	97.80-99.00
Ophthalmologist screening [24]	94.67	94.57-97.43
7. Compliance of screening [33], %	86.00	-

*DR* Diabetic retinopathy, *VTDR* Vision-threatening diabetic retinopathy

**Table 2:** Health system and Societal Costs Per Person for DR Screening and Treatment in US Dollars

Cost Items	Cost ($)	Sensitivity Analysis Range
**Health System costs**		
1. Screening		
AI software	1.447	0.723–2.17
Ophthalmologist salary	3.213	1.606–4.819
Eye examination	1.63	0.815–2.445
2. Follow-up visit		
Follow-up examination	2.199	1.099–3.298
3. Laser treatment	347.177	173.589–520.766
**Societal costs**		
1. Income loss		
Blindness (for the first year) [34]	8,920	4,460–13,380
Blindness (in the following years) [34]	3,600	1,800–5,400
Screening	3.18	1.59–4.77
Treatment	12.7	6.35–19.05
2. Transportation [35]		
Screening	0.58	0.29–0.87
Follow-up visit	2.30	1.15–3.45

AI screening costs include cost of AI software and eye examination while ophthalmologist screening costs include salaries of ophthalmologist and eye examination. The costs of AI screening group’s follow-up visit include cost of AI software and follow-up examination. The costs of ophthalmologist screening group’s follow-up visit include cost of ophthalmologist salaries and follow-up examination

### Costs

Costs were estimated from the health system perspective and societal perspective. Costs were collected in Chinese Yuan and then converted into US dollars at an exchange rate of 6.9129 yuan per dollar in 2020, as shown in Table 2.

From the societal perspective, the costs included direct costs (medical and nonmedical) and indirect costs (i.e., work time lost). We estimated health system cost of only direct medical costs. Direct medical costs can be divided into three parts: (1) costs of screening, (2) costs of follow-up examinations, (3) costs of laser treatment. For AI screening, the costs of screening included AI software fee, salaries of eye care professionals, maintenance of equipment, advertising and building rent. For ophthalmologist screening, the costs of screening included salaries of eye care professionals, maintenance of equipment, advertising, building rent, as well as salaries of ophthalmologists. Direct nonmedical costs consisted of transportation fees related to visits to the community hospitals and superior hospitals. Indirect costs consisted of the monetary value of work time lost, which was spent on screening, follow-up examinations and laser treatment. The costs caused by blindness were derived from a study by Tang et al. [34], which consisted of 53.2% direct medical costs, 6.4% direct nonmedical costs, and 40.4% indirect costs. Indirect costs included loss of labour resources, loss of productivity among caregivers and modification costs. The total cost for the first year of blindness was $8,920 and only indirect costs (i.e., $3,600) are incurred in subsequent years until death. Costs were discounted at an annual rate of 3%.

### Effectiveness

Effectiveness was measured with quality-adjusted life years (QALYs) gained. Utility weights of different DR states were obtained from the published literature, using time trade-off method [30]. The QALYs were calculated by multiplying the utility values and the time spend in this health state [36]. The QALYs were also discounted at an annual rate of 3%.

### Cost-effectiveness analysis

We analyzed the cost-effectiveness of the two screening strategies by using the Markov model. If the cost of AI screening was less expensive but provided more effectiveness than ophthalmologist screening, the ophthalmologist screening was dominated. Compared with no screening, we estimated the ICER of AI screening as the difference between the costs divided by the difference between the total QALYs gained. We determined whether AI screening was cost-effective by comparing the ICER with the threshold suggested by World Health Organization, 1–3 times the per capita gross domestic product (GDP), which was considered cost-effective [37]. The per capita GDP of China in 2019 was $10,255.03.

### Sensitivity analysis

We performed a one-way sensitivity analysis in which parameters varied once at a time over the estimated ranges presented, to evaluate the impact of the uncertainty of some key model parameters on ICER. The minimum and maximum values were estimated from 95% confidence intervals for mortality multipliers, transition probabilities, utility values. For costs, a range of ± 50% was applied. The discount rate range we used was recommended by WHO, 0%-6% [38]. Additionally, we performed a probabilistic sensitivity analysis in which variables varied simultaneously. It took repeated 10,000 samples across the ranges of the parameters. The results were presented graphically as cost-effectiveness curve, which was used to show the proportion of iterations in which AI screening was cost-effective at different willingness-to-pay thresholds.

## Results

### Cost-effectiveness analysis

**Fig. 2:**
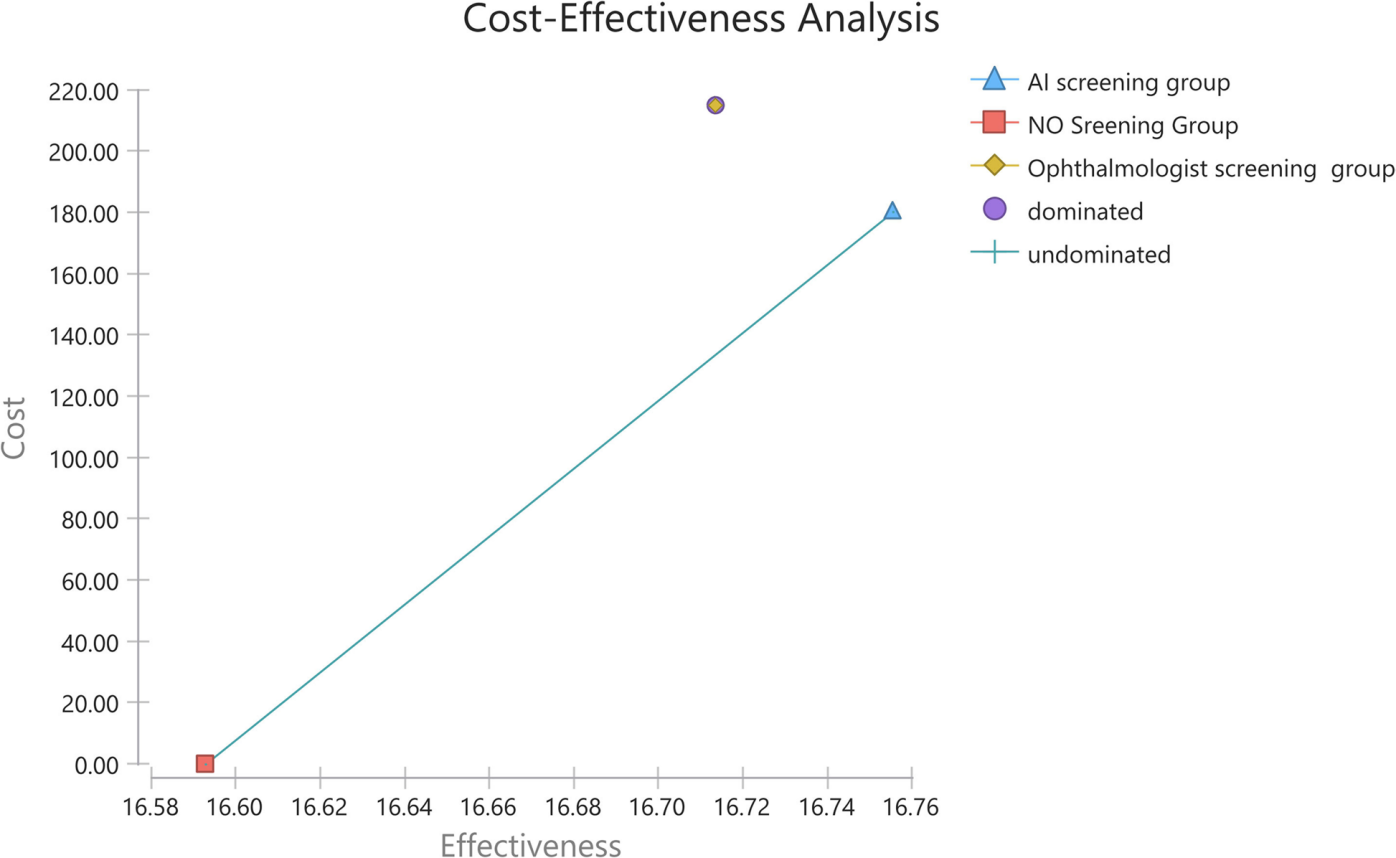
Cost-effectiveness curve showing dominated strategies and undominated strategies under the health system perspective

The model estimated the cost and health outcomes of the two screening groups. The cost-effectiveness results, from the health system perspective in the 35 cycles, are shown in Fig. 2 and Table 3. Relative to no screening, AI screening was more expensive with an incremental cost of $180.19, but more effective with an incremental QALYs of 0.16. The ophthalmologist screening was more expensive (incremental costs of $34.86) and less effective (incremental QALYs of -0.04) compared with the AI screening. The ICER of AI screening compared with no screening group was $1,107.63/QALY gained, less than the threshold of $30,765.09, which was three times Chinese per capita GDP in 2019. AI screening was considered cost-effective. Figure 2 showed that the ophthalmologist screening group was dominated by AI screening.

**Table 3:** Cost-effectiveness results from the health system and societal perspectives

	Cost ($)	Incremental Cost ($$\Delta$$$)	Effectiveness (QALYs)	Incremental Effectiveness ($$\Delta$$ QALY)	ICER ($$\Delta$$$/$$\Delta$$ QALY)
1. Health system perspective					
No screening	0	-	16.59	-	-
AI screening	180.19	180.19	16.76	0.16	1,107.63
Ophthalmologist screening	215.05	34.86	16.71	-0.04	Dominated
2. Societal perspective					
No screening	0	-	16.59	-	-
AI screening	1,683.23	1,683.23	16.76	0.16	10,347.12
Ophthalmologist screening	1,775.48	92.25	16.71	-0.04	Dominated

*QALY* Quality-adjusted life year, *ICER* Incremental cost-effectiveness ratio

**Fig. 3:**
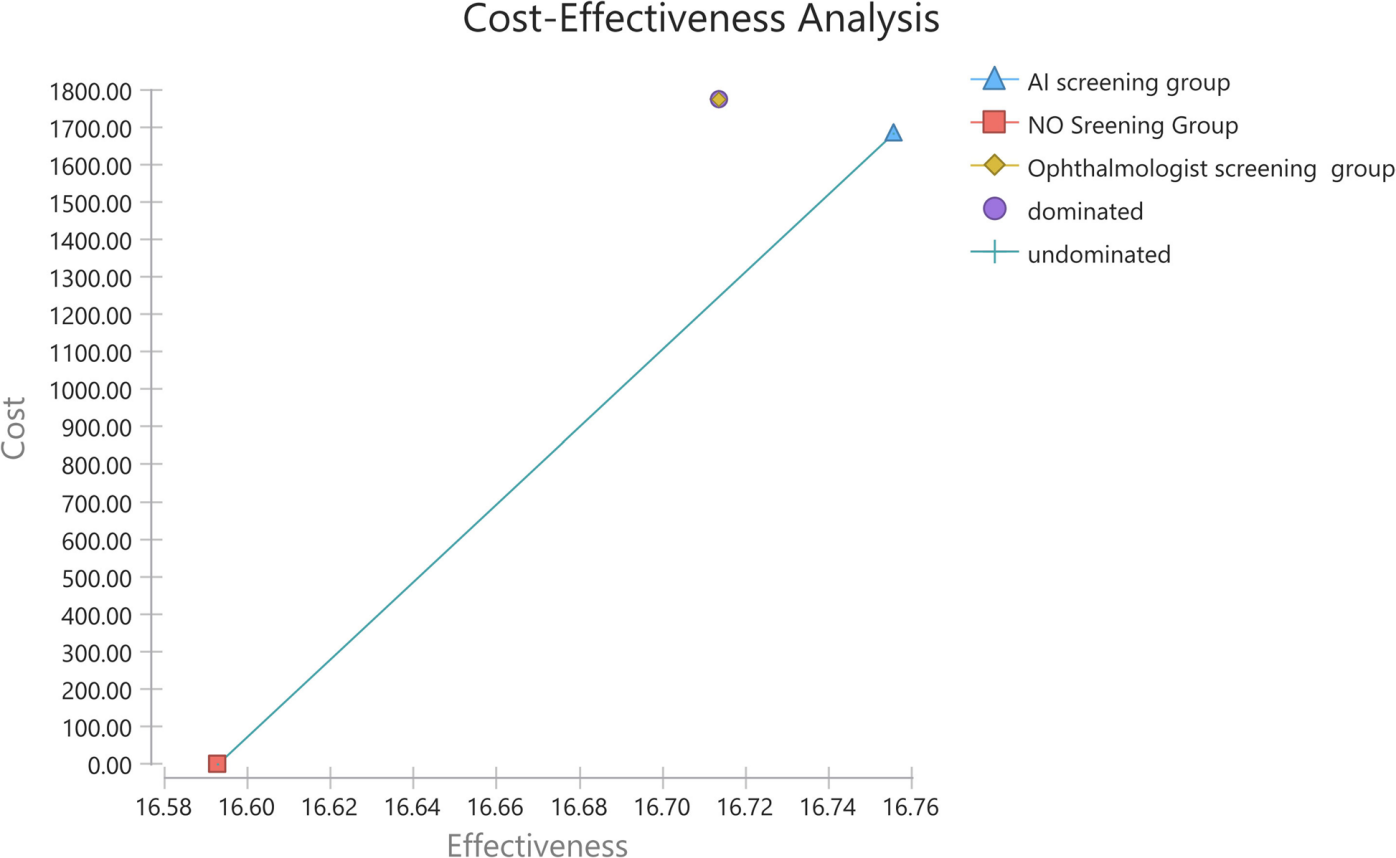
Cost-effectiveness curve showing dominated strategies and undominated strategies under the societal perspective

The ophthalmologist screening was still dominated by AI screening, from the societal perspective, as shown in Fig. 3 and Table 3. AI screening costs less than ophthalmologist screening ($1,683.23 versus $1,775.48). Relative to no screening, the ICER of AI screening was $10,347.12, below the cost-effective threshold ($10,255.03-$30,765.09). The ophthalmologist screening was more expensive (incremental costs of $92.25) and less effective (incremental QALYs of -0.04) compared with the AI screening. So AI screening was more cost-effective compared with ophthalmologist screening from both health system perspective and societal perspective.

### Sensitivity analysis

#### One-way sensitivity analysis

**Fig. 4:**
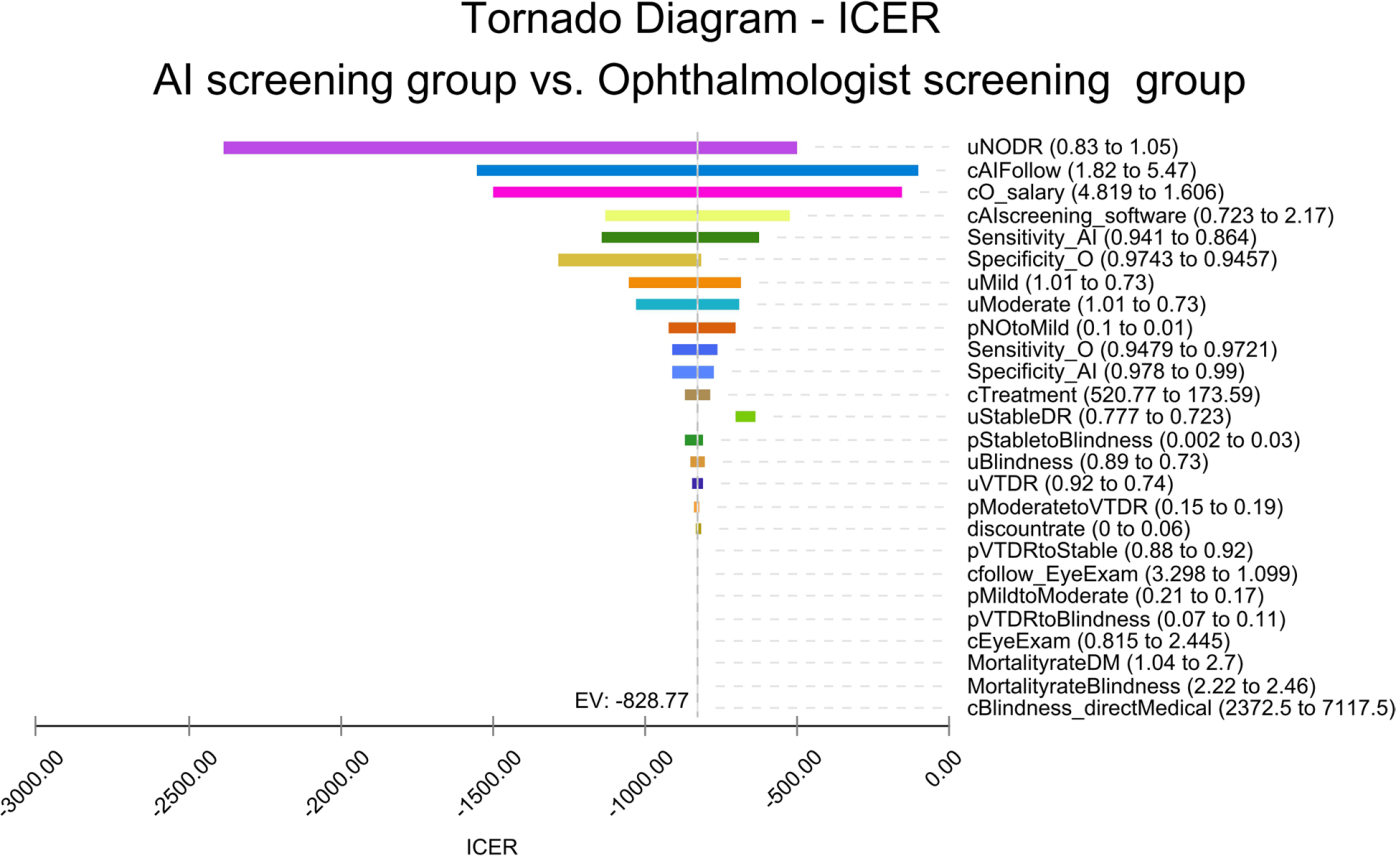
One-way sensitivity analysis (Tornado diagram) under the health system perspective. Legend: c=cost; AI=AI screening; o=ophthalmologist screening; p=transition probabilities; u=utility; ICER=incremental cost-effectiveness ratio; DR=diabetic retinopathy; VTDR=vision-threatening diabetic retinopathy; DM=diabetes mellitus

**Fig. 5:**
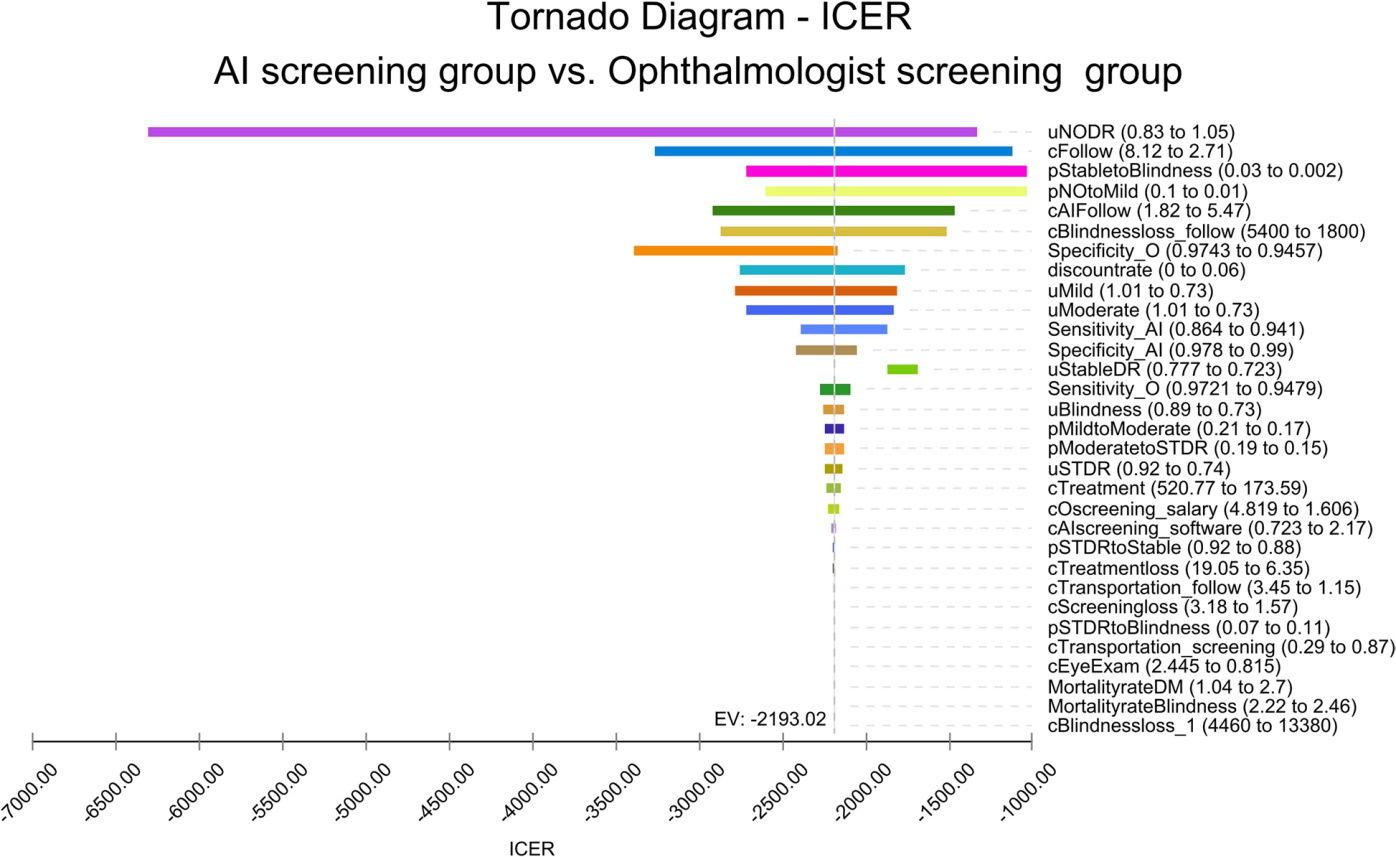
One-way sensitivity analysis (Tornado diagram) under the societal perspective. Legend: c=cost; AI=AI screening; o=ophthalmologist screening; p=transition probabilities; u=utility; ICER=incremental cost-effectiveness ratio; DR=diabetic retinopathy; VTDR=vision-threatening diabetic retinopathy; DM=diabetes mellitus

The results of the one-way sensitivity analysis in the Tornado diagram from the health system perspective and societal perspective are shown in Fig. 4 and Fig. 5. The one-way sensitivity analyses revealed the effect which the model variables had on the results when other model variables remained unchanged. From the health system perspective, the most influential parameter was the utility of NO DR, followed by the costs of ophthalmologist salaries. From the societal perspective, the most influential parameter was still the utility of NO DR, followed by the costs of follow-up visit of ophthalmologist screening.

### Probabilistic sensitivity analysis

**Fig. 6:**
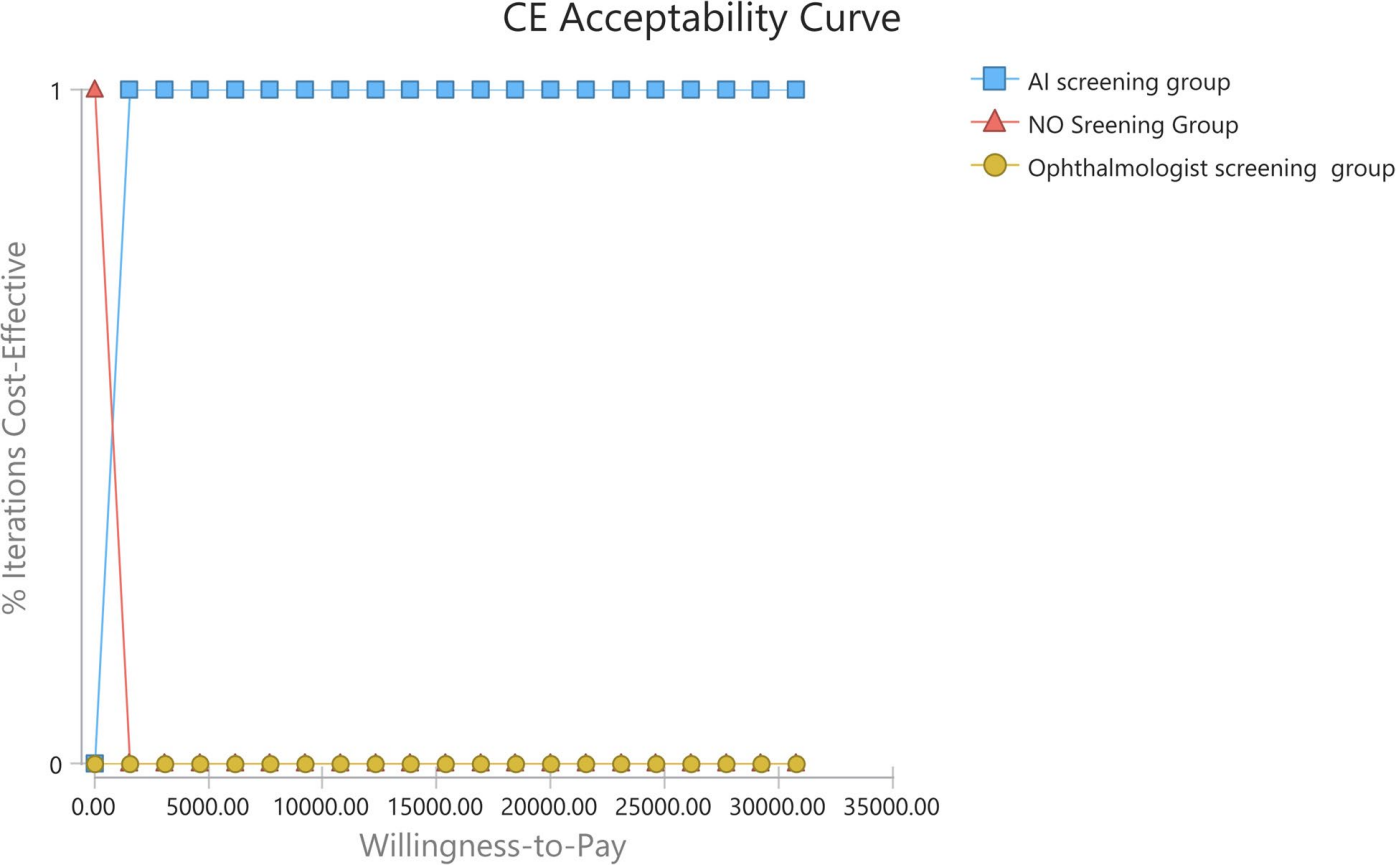
Cost-effectiveness acceptability curve under the health system perspective

The cost-effectiveness acceptability curve from the probabilistic sensitivity analysis (PSA) under the health system perspective was shown in Fig. 6. The ophthalmologist screening was considered cost-effective in 0 iterations at any given willingness-to-pay value. It showed AI screening was cost-effective versus no screening and ophthalmologist screening in 100% of the iterations at the willingness-to-pay threshold of $30,765.09/QALY, 3 times Chinese per capita GDP in 2019, under the health system perspective. The mean costs of non-dominated strategies, no screening and AI screening, were $0 and $180.19, respectively. The mean QALY of no screening was 16.59 QALYs and that of AI screening was 16.76 QALYs. The PSA results showed that the ICER between non-dominated strategies would be $1,107.63/QALY gained, which was below the threshold.

**Fig. 7:**
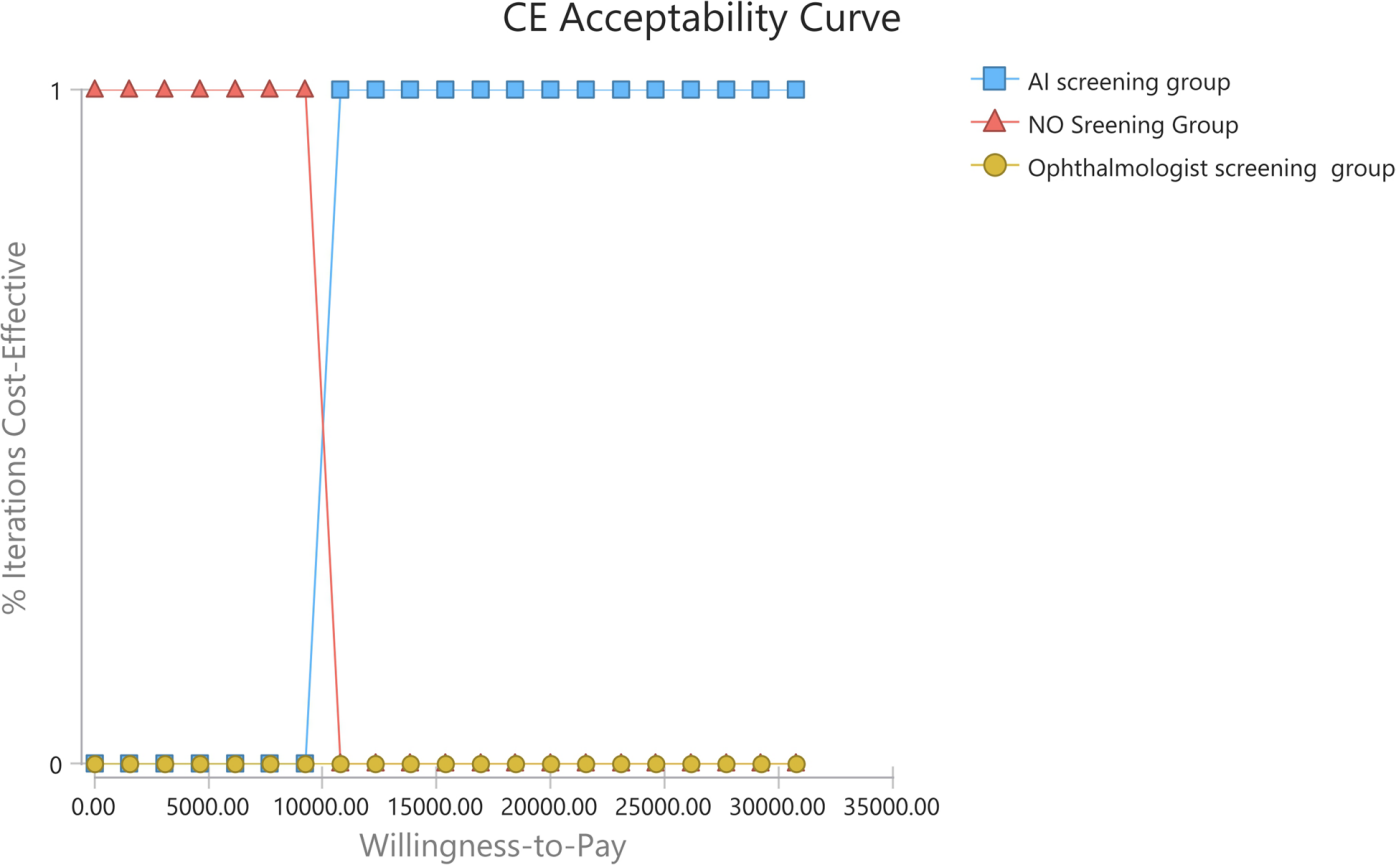
Cost-effectiveness acceptability curve under the societal perspective

From the societal perspective, the ophthalmologist screening was considered cost-effective in 0 iterations at any given willingness-to-pay value as shown in Fig. 7. It showed AI screening was more cost-effective compared with no screening and ophthalmologist screening in 100% of the iterations at the willingness-to-pay threshold. The mean costs for AI screening and ophthalmologist screening were $1,683.23 and $1,775.48 respectively. The mean QALY of ophthalmologist screening was 16.71 QALYs and that of AI screening was 16.76 QALYs. Compared with no screening group, the ICER of AI screening, the dominant strategy, was $10,347.12, below the cost-effective threshold.

## Discussion

This model-based economic evaluation compared two DR screening strategies from the health system perspective and societal perspective. The results suggested that AI screening would be the most cost-effective compared with no screening and ophthalmologist screening based on the threshold, 1–3 times per capita GDP of Chinese in 2019. Base-case results indicated that AI screening generated a cost saving of $34.86 while generating more QALYs (incremental QALYs of 0.04) relative to ophthalmologist screening from the health system perspective. From the societal perspective, AI screening generated a cost saving of $92.25. Promotion across the country can save labor costs and resources, as well as reduce the occurrence of DR. Our results suggested that the adoption of AI screening at the community health service stations was economically sound.

The lower cost of AI screening relative to ophthalmologist screening is attributed to the difference in the cost of grading fundus images. In our study, the costs of AI screening and ophthalmologist screening were basically the same except for the costs of grading. It costs less to grade a fundus image by AI screening relative to ophthalmologist screening ($1.447 per patient versus $3.213 per patient). This is the main reason causing the difference between the cost-effectiveness of the two screening strategies.

Our results consist with the findings in previous literatures although the research settings are different [39–42]. The study of Tufail et al. reported the cost saving to be 12% to 21% for DR screening in the United Kingdom using ML (an AI-based technology) in comparison with human assessors [39]. A Scottish study showed a 46.7% cost-reduction by replacing first-level human assessment with automated grading in a national DR screening program [40]. The study by Xie et al. from Singapore reported fully automated DLS (deep learning systems) had a cost savings of 14.3% compared with human assessment system [41]. The study of Fuller et al. reported the primary care-based ARIAS screening among low-income patients with diabetes is substantially less costly [42].

Our study applied a more comprehensive system of prognosis after people were diagnosed with diabetes, based on Markov model. In our study, health states included DR states, blindness, death and the stable state after laser treatment, which reflect the natural progression of DR. We took more factors into consideration in our cost estimation. We calculate age-dependent mortality by using linear interpolation in order to obtain an accurate outcome.

We also perform a one-way sensitivity analysis and probabilistic sensitivity analysis to assess the uncertainty of cost and effectiveness. In our study, the results of cost-effectiveness analysis from two perspectives helped to provide more convincing and well-rounded evidence about the cost-effectiveness of AI based DR screening for the decision-making agency.

### Study limitations

First, the transition probabilities and the utility values were partly derived from the results in other countries, which might be not exactly consistent with those in China, resulting in potential uncertainty in our study. We think the data we used were best available for our analysis. Second, we assumed that the patients’ compliance in AI screening and ophthalmologist screening were the same. Actually, as a cost- and time-saving strategy, AI screening is supposed to be more acceptable compared with ophthalmologist screening. Moreover, we assumed the compliance of follow-up examination and laser treatment were 100% to simplify the calculation. Third, we didn’t consider the rate of fundus images that could not be graded accurately, and just from one study to determine the sensitivity and specificity of the AI screening. Fourth, in our study, we compared the ICER with the per capita GDP of the whole country instead of rural China. Fifth, we assumed all patients with newly diagnosed diabetes had no DR. In fact, some patients have a relative long duration of diabetes before definite diagnosis were established and early-stage diabetic retinopathy might occur.

Our analysis highlights the great need for further research in the areas of AI screening for DR. The distribution of different DR stages in rural China as well as DR progression rates between different stages should be surveyed analyzed. Additionally, the data on detailed costs for AI screening conducted in rural China, especially indirect costs (i.e., the income loss of patients’ family associated with their blindness) and the compliance of screening and follow-up examination should be investigated. Moreover, screening intervals have been found to have influence on cost-effectiveness in many countries [43–47]. Since we used screening intervals recommended by ICO guidelines for diabetic eye care, individualized screening intervals suitable for Chinese patients should be investigated.

To the best of our knowledge, this is the first economic evaluation of AI-based screening for DR in rural China. The results that AI screening is cost-effective compared with conventional screening indicate that AI might be a promising strategy in the future. Considering the lack of medical resource and high incidence of DR in rural area of China, we think that wide application of AI screening might improve the current situation. The findings by Lian et al. showed that free DR screening was more cost-effective for a healthcare provider than paying screening. Charging a small co-payment will decrease the willingness of the potential DR patients to participate in screening, especially the low-income subjects [48]. Due to the large amount of the population in the rural China and limited healthcare budget, free DR screening is not a practical and feasible approach at present. With the rapid development in AI technology, the cost of AI-based DR screening is expected to decrease dramatically and the performance will be further improved. Additionally, the AI screening will greatly alleviate the issue of ophthalmologists’ shortage in rural China. Meanwhile, since many oversea countries are faced with similar problems, such as lack of medical facilities, expensive manual screening costs and limited access to conventional screening programs for DR screening, AI screening for diabetic retinopathy may also be a feasible solution.

## Conclusions

The results of our study show that AI screening saves costs in comparison with ophthalmologist screening for DR, which provide evidence for extending the application of AI-based DR screening across rural China.

## Data Availability

The datasets generated or analyzed during the current study are not publicly available due to privacy restrictions but are available from the corresponding author on reasonable request.

## Abbreviations

DR: Diabetic retinopathy
AI: Artificial intelligence
ICER: Incremental cost-effectiveness ratio
QALYs: Quality-adjusted life years
GDP: Gross domestic product
DLS: Deep learning systems
VTDR: Vision-threatening diabetic retinopathy
NPDR: Non-proliferative diabetic retinopathy
PSA: Probabilistic sensitivity analysis
